# Investigation of the Critical Behavior, Magnetocaloric Effect and Hyperfine Structure in the Fe_72_Nb_8_B_20_ Powders

**DOI:** 10.3390/ma13204476

**Published:** 2020-10-09

**Authors:** Safia Alleg, Thaounza Chabi, Nadia Bensebaa, Joan Saurina, Lluisa Escoda, El-Kebir Hlil, Joan-Josep Suñol

**Affiliations:** 1 Laboratoire de Magnétisme et Spectroscopie des solides (LM2S), Département de Physique, Université Badji Mokhtar Annaba, BP12, 23000 Annaba, Algeria; safia_alleg@yahoo.fr (S.A.); thaounza@yahoo.fr (T.C.); n_bensebaa@yahoo.fr (N.B.); 2 Department Física, Universitat de Girona, Campus Montilivi s/n, 17003 Girona, Spain; joan.saurina@udg.edu (J.S.); lluisa.escoda@udg.edu (L.E.); 3 Institut Néel, Grenoble INP, Université Grenoble Alpes, CNRS, 38000 Grenoble, France; El-Kebir.Hlil@neel.cnrs.fr

**Keywords:** ball milling, Fe-Nb-B system, magnetocaloric properties, thermal analysis, Mössbauer spectroscopy, critical behavior

## Abstract

Microstructure as well as magnetic, thermal and magnetocaloric properties of the mechanically alloyed Fe_72_Nb_8_B_20_ powders have been investigated by means of Mössbauer spectrometry, differential scanning calorimetry (DSC), and magnetic measurements. The Mössbauer spectrometry results showed the formation of nanostructured Fe(B) and Fe(Nb) solid solutions, Fe_2_B boride, and an amorphous phase. The endothermic and exothermic peaks that are observed in the DSC curves might be related to the Curie temperature, and the crystallization of the amorphous phase, respectively. The critical exponent values around the magnetic phase transition of the amorphous phase (T_C_ = 480 K), are deduced from the modified Arrott plots, Kouvel−Fisher curves and critical isotherm examination. The calculated values (β = 0.457 ± 0.012, γ = 0.863 ± 0.136 and δ = 3.090 ± 0.004) are near to those of the mean field model, revealing a dominating role of magnetic order arising due to long-range ferromagnetic interactions, as the critical exponents are mean-field-like. The maximum entropy change and the refrigerant capacity values are 1.45 J/kg·K and 239 J/kg, respectively, under a magnetic field of 5 T.

## 1. Introduction

Magnetic refrigeration (MR) is a promising alternate to the conventional refrigeration, and a developing technology that enhances energy efficiency and environmental respect as it uses clean energy. MR is established on the magnetocaloric effect (MCE) which represents a thermal reaction or a temperature variation of certain magnetic solids under the solicitation/removal of a magnetic field in an adiabatic condition [1]. Indeed, the application of a magnetic field gives rise to the alignment of the magnetic moments of a solid parallel to it and hence, to the increase of the temperature owing to the released thermal energy. Consequently, the magnetic entropy is reduced. By removing the magnetic field, the sample cooled down due to the random orientation of the magnetic moments, and the entropy increased [2].

Many researches have been devoted to nanoscale magnetic materials magnetic materials owing to a large MCE in the superparamagnetic nanostructured materials [3,4]. According to their magnetic phase transition, magnetic refrigerant materials can exhibit either a first order magnetic phase transition (FOMT) or a second order magnetic phase transition (SOMT). The former is described by large magnetic entropy variations, considerable hysteresis, a narrow temperature range, and a strong correlation between magnetism and crystallographic structure [5,6]. Whereas the SOMT materials show no structural transition at the Curie temperature ($T_{C}$) that could improve the magnetization change, and they have negligible hysteresis, lower magnetic entropy change peaks and a wide temperature range [7,8,9,10]. The main problem of magnetic phase transitions theory consists in studying the behavior of a given system in the neighborhood of the ferromagnetic (FM) to paramagnetic (PM) magnetic transition temperature. Indeed, some physical magnitudes corresponding to the system have singularities at the critical point. These singularities are, generally, expressed in terms of power law categorized by critical exponents, which qualitatively determine the nature of the behavior of a given system. According to different theoretical models [11], the magnetic phase variation near $T_{C}$ is defined by a set of critical exponents (β, γ and δ), where β is related to the spontaneous magnetization Ms (μ_0_H = 0) under $T_{C}$; γ is linked to the initial magnetic susceptibility χ_0_ below $T_{C}$, and δ can be deduced from the magnetic isotherm at $T=T_{C}$.

Fe-Nb-B alloys are very stimulating materials owing to their soft magnetic properties (high magnetization of saturation, low core losses, zero magnetostriction, etc.) that can be achieved after optimum thermal heat treatment [12]. Accordingly, they have many industrial applications such as telecommunications, magnetic heads, sensors, power transformers, etc. [13,14]. Besides, Fe-Nb-B alloys exhibit other possible functionalities such as MCE [15,16]. Indeed, in the amorphous Fe_93-x_Nb_7_B_x_ (x = 9, 14 and 20) prepared by rapid quenching, the entropy values are $-\Delta S_{M}\;=1.44,\;1.07\;\mathrm{and}\;0.97\;J/\mathrm{kg}\cdot K$ for x = 9, 14 and 20, respectively [17]. The temperature dependence of the MCE was studied in amorphous and nanocrystalline Fe_80.5_Nb_7_B_12.5_ melt-spun ribbons [18]. The maximum entropy change was about 0.72 J/kg·K, at $T_{C}\;\sim \;363\;K$ of the amorphous phase, upon a magnetic field modification of 0.7 T. Nevertheless, the magnetic entropy variation decreased and its peak broadened with the progressive nanocrystallization of the amorphous ribbons. 

Many methods have been used to produce magnetocaloric materials such as mechanical alloying (MA) [19,20], solid state reactions [21], sol gel routes [22], melt spinning [23,24], etc. In the mechanically alloyed powders, the MCE response can be affected by several factors such as the alloy composition, the multiphase character, the demagnetizing field effect, etc. [25]. Consequently, depending on the experimental procedure, the obtained results might be different. Hence, the goal of the current work was to study the structure and the magnetocaloric, magnetic and thermal properties of the ball-milled Fe_72_Nb_8_B_20_ powders. The critical behavior near the Curie temperature of the amorphous phase is also discussed.

## 2. Experimental Details 

Fe_72_Nb_8_B_20_ (wt. %) powders were ball-milled for 50 h. The experimental details are reported in reference [26]. The local ^57^Fe environment was studied by Mössbauer spectrometry in transmission geometry, at 300 K, by means of a ^57^Co source diffused in an Rh matrix. Setaram DSC131 evo apparatus (DSC) (Setaram Instrumentation, Lyon, France) was used to examine the thermal behavior within the temperature range 323−973 K, under argon atmosphere, by using different heating rates. The hysteresis loops were measured, at room temperature, with a Lakeshore 7404 vibrating sample magnetometer (VSM) (LakeShore, Westerville, Oh, USA) under an applied magnetic field of 1.5 T. Magnetization versus temperature comparisons were performed on a BS2 magnetometer developed at the Néel Institute. The demagnetization field effect might have been neglected because the sample was used in powder form. The demagnetization factor D = 0.027 was determined from the slope of the M(H_app_) curve near zero field. The corrected magnetic field after subtraction of the demagnetization field is $\;H=H_{app}-H_{D}=H_{app}DM$, where H_app_ is the applied magnetic field.

## 3. Results and Discussions

### 3.1. Hyperfine Structure

**Figure 1:**
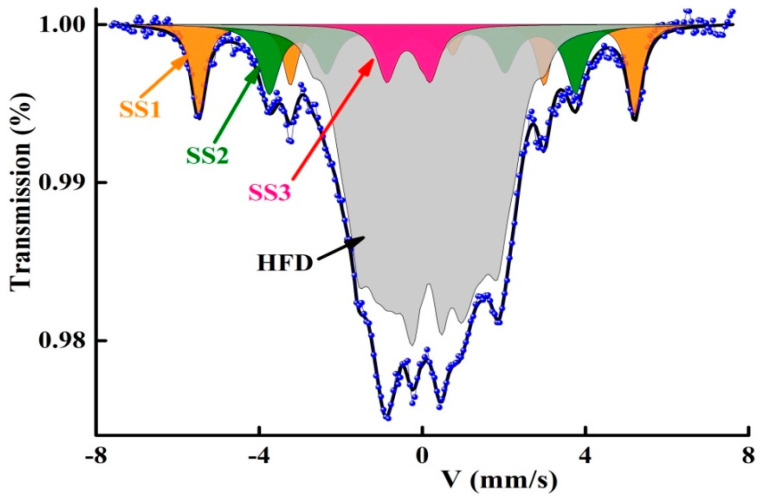
Fitting of the Mössbauer spectrum with four components (SS1, SS2, SS3 and HFD).

Mössbauer spectrometry of ^57^Fe enables the examination of the iron sites by the determination of the local Fe environment, composition variations and the spreading of Fe atoms. The coexistence of crystalline and amorphous magnetic phases is evidenced by the presence of sharp and widened magnetic lines, respectively, in the Mössbauer spectra of the Fe_72_Nb_8_B_20_ powders (Figure 1). The presence of nonmagnetic B and/or Nb atoms in the neighborhood of Fe atoms leads to the atomic disorder which is manifested by the enlargement of the Mössbauer lines. In order to identify the different Fe sites, the Mössbauer spectra were fitted by a least-squares MOSFIT program [27], by using two magnetic sextets (SS1, SS2), one paramagnetic doublet (SS3) and a hyperfine field distribution (HFD). The obtained hyperfine parameters magnetic field (B_hf_), isomer shift (IS), quadrupolar splitting/shift (QS/2ε), line width (Γ) are presented in Table 1. The IS is related to α-Fe at room temperature.

The hyperfine parameters of the sextet SS1 (B_hf_ = 33 T and IS = 0.011 mm/s) can be associated with an Fe-rich Fe(B) solid solution containing a very small concentration of boron atoms. Indeed, existence of one B atom as the nearest neighbor (nn) of an Fe atom raises IS by about 0.07 mm/s. In such Fe(B) sites, the average number of B atoms (n_B_) can be estimated from the linear relationship between the hyperfine magnetic field (B_hf_) and the number of B atoms [28]: B_hf_ = 33.6–2.7n_B_. Accordingly, the number of B atoms in the sextet SS1 is about n_B_ = 0.22. The sextet SS2 with B =23.2 T and IS = 0.062 mm/s, is ascribed to the Fe_2_B boride phase. The paramagnetic doublet with IS = −0.203 mm/s and QS = 0.996 mm/s can be linked to an Fe(Nb) solid solution, since the existence of one Nb atom as the first or second nn of an Fe atom diminishes IS by 0.04 mm/s [29]. The HFD is due to the existence of numerous non−equivalent Fe surroundings where the Fe atoms are mainly surrounded by B atoms in their neighborhoods. The HFD can be linked to a B-rich FeB amorphous matrix. These results agree well with XRD findings (not shown here) [26].

### 3.2. Thermal Analysis

**Figure 2:**
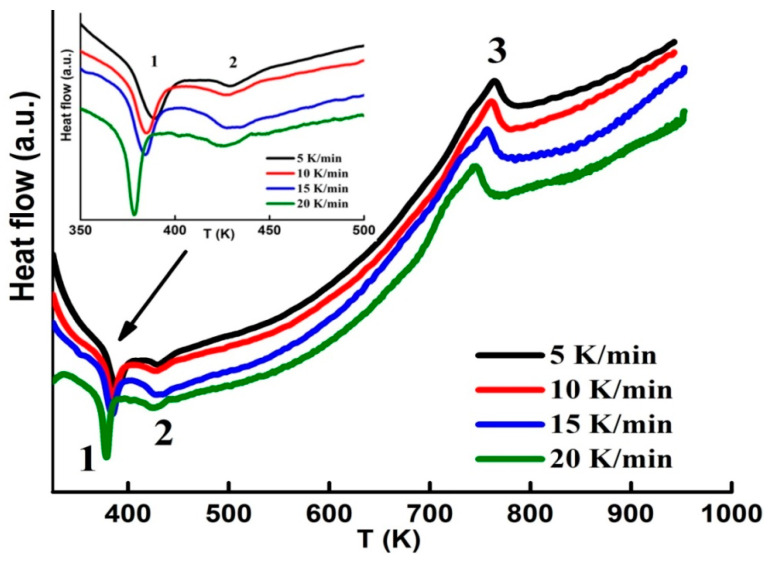
DSC scans measured with several heating rates (the inset shows the first peak). Peaks 1 and 2 correspond to two FM-PM transitions, and peak 3 to the crystallization process.

The continuous heating DSC curves with several heating rates (5, 10, 15 and 20 K/min) are shown in Figure 2. The DSC scans exhibit two endothermic peaks at about ~389 K and 428 K that can be associated to the magnetic transition (T_C_) of the amorphous phase, since those of α−Fe (1043 K) and Fe_2_B (1015 K) are higher [30]. The existence of two T_C_ might be related to the impurity phases and/or the distribution of Curie transitions in the highly disordered amorphous matrix. Similar results have been observed in other ball-milled powder alloys [31,32]. The obtained values are analogous to those of B containing alloys [17,18]. The broad exothermic peak in the temperature range 650−800 K can be attributed to the crystallization of the amorphous phase. The apparent activation energy under continuous heating conditions can be calculated by means of the Kissinger peak displacement method [33]: ln(β/T^2^) = −E_A_/RT + const., where β is the constant heating rate, R is the gas constant and T stands for the crystallization peak temperature. The activation energy E_A_ = 342 ± 10 kJ/mol has been estimated from the linear fit of ln(β/T^2^) versus 1/T plot. This value can be linked to a grain growth process. A slightly different value of about 324 ± 35 kJ/mol has been found in the 80h ball-milled Fe_74_Nb_6_B_20_ powders [34]. Those discrepancies might be related to the milling conditions and the obtained phases. 

### 3.3. Magnetic Properties 

**Figure 3:**
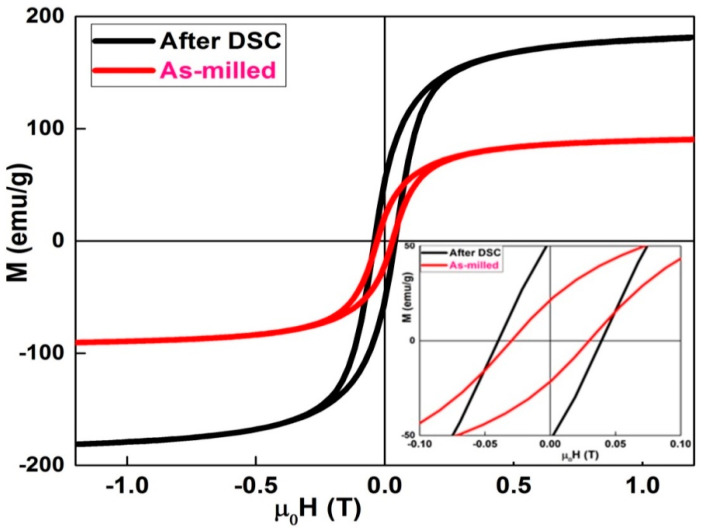
Hysteresis cycles of the as-milled and heat treated Fe_72_Nb_8_B_30_ powders.

Figure 3 displays the hysteresis loops recorded at room temperature for the ball-milled and heat-treated powders after DSC analysis. The hysteresis loops show the same trend. They are saturated and exhibit a sigmoidal shape type. For the as-milled powders, the coercivity is 0.0302 T and the saturation magnetization is 92 emu/g. However, after DSC heat treatment, both the coercivity and saturation magnetization increased to ~0.0397 T and 181 emu/g, respectively. The increase in coercivity may be attributed to a higher number of non-magnetic phases and/or Fe_2_B boride. However, the increase in saturation magnetization might be correlated to the formation of α−Fe nanocrystals. 

**Figure 4:**
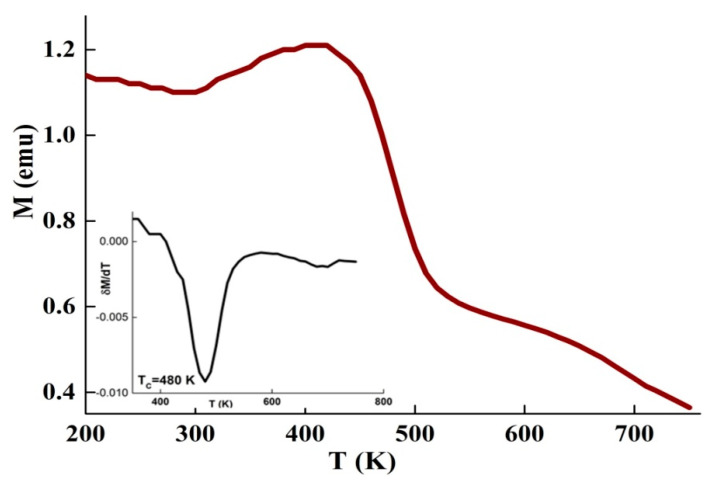
Evolution of the magnetization against the temperature under H= 0.05 T. The derivative of δM/δT is presented in the inset.

Figure 4 displays the magnetization as a function of temperature, M(T), measured in a magnetic field of 0.05 T. T_C_ of the amorphous phase that corresponds to the minimum of δM/δT, was found to be 480 K. This value is higher than that observed in the DSC curves by about 100 K. The measured T_C_ depends on the compositional heterogeneity, strain distribution, sample shape and/or the determination method, in particular in several constituent alloys [35]. During heating, the sensitivity of a reaction is related to its energy evolved as well as to the mass of the sample. In the M(T) curve, T_C_ is usually determined from the drop of magnetization or the inflection point method, whereas, DSC detects T_C_ as a heat flow variation owing to the small quantity of energy accompanying the ferromagnetic-to-paramagnetic phase transition. Hence, the endothermic reaction that happens below T_C_ represents the absorbed energy during heating to induce randomization of the magnetic dipoles. Furthermore, the presence of many phases should impact the modification of the magnetization around T_C_. This later depends on the exchange interaction between the magnetic moments, which in turn depends on the distance between the magnetic atoms. Consequently, T_C_ is dependent on the composition of the amorphous phase. For example, in the Fe_80.5_Nb_7_B_12.5_ melt-spun ribbons $T_{C}\;$ was found to be 363 K [18]. In the amorphous Fe_100-x_B_x_ alloys (10≤ x ≤35 at. % B), $T_{C}$ of the amorphous phase increased with the augmentation of the boron content from 480 K for x = 10 up to 820 K for x = 28, and then decreased [36]. 

### 3.4. Magnetocaloric Effect

**Figure 5:**
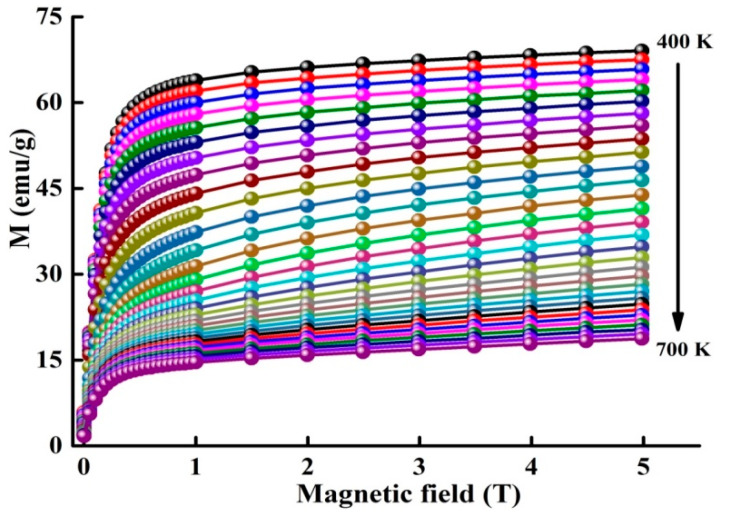
Isothermal magnetization curves around $T_{C}$ at different temperatures.

Figure 5 displays the isothermal M(H) plots in the temperature range 400−700 K. The magnetocaloric behavior can be studied through the evaluation of the magnetic entropy changes ΔS_M_ from the magnetization measurements by using the Maxwell Equation:

(1) $$\Delta S_{M}\left(T,\Delta H\right)=S_{M}(T,H_{2})-S_{M}\left(T,H_{1}\right)=\int_{H_{1}}^{H_{2}}{\left(\frac{\partial M}{\partial T}\right)}_{T}dH$$

**Figure 6:**
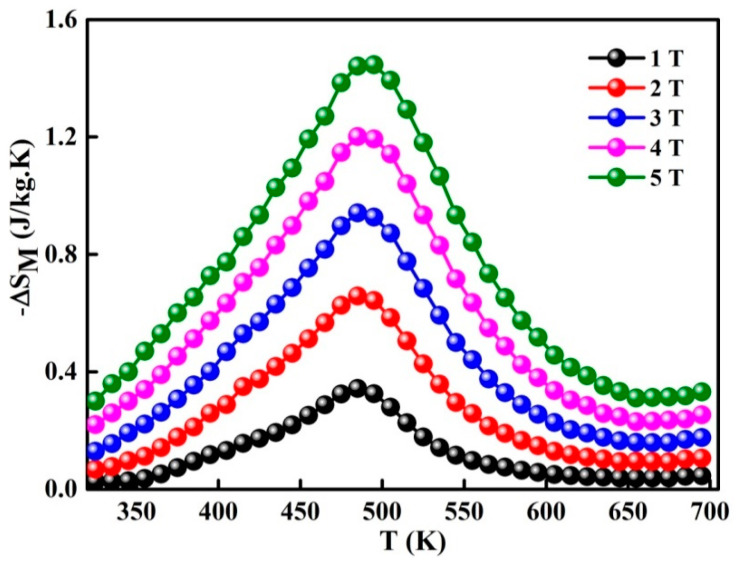
Temperature dependence of change in magnetic entropy for different magnetic fields.

With $H_{1}$ and $H_{2}$ the applied magnetic fields where $H_{1}<H_{2}$, and $\Delta H=H_{2}-H_{1}$. The numerical Maxwell’s Equation can be given by:

(2) $$\Delta S_{M}\left(T,M\right)=\underset{i}{\sum }\frac{M_{i+1}\left(T_{i+1},H_{i+1}\right)-M_{i}\left(T_{i},H_{i}\right)}{T_{i+1}-T_{i}}\;\Delta H$$

where $M_{i}$ and $M_{i+1}$ are the experimental data of the magnetization at $T_{i}$ and $T_{i+1}$, respectively, under the magnetic field H_C_, the temperature dependence of change in magnetic entropy − ΔS_M_(T) is presented in Figure 6. The magnetic entropy change versus temperature shows a peak, which has been previously identified as $T_{C}$. One also observes that ΔS_M_ increases as the applied magnetic field rises and attains 1.45 J/kg·K under 5 T. Different values of −ΔS_M_ and $T_{C}$ (Table 2) are obtained for certain Fe-Nb-B alloys [17,18,37,38,39]. Those differences might be accredited to the experimental conditions such as the fabrication method, alloy composition, particle size and shape, structure, phase nature, matrix interactions, neighboring particles, etc. Those parameters have a deep effect on the magnetic behavior of a material.

A linear dependence has been found between the maximum entropy change and log(H). The Equation is:

(3) $$\Delta S_{M}\left(H\right)=n\log H+C$$

The linear fitting is $\Delta S_{M}\left(H\right)=1.1192\log H+0.3386$ (R^2^ = 0.9694). This tendency indicates that when increasing the applied magnetic field, the maximum entropy change increases (with a factor below that corresponding to magnetic field change).

The refrigerant capacity (RC) associated with the entropy variation represents a way to evaluate the magnetocaloric efficacy of materials. RC denotes the transferred quantity of warmth between the warm and cold tanks [40]. RC is determined experimentally from ΔS_M_(T) and the full width at half maximum (δT_FWHM_) of the peak entropy, since it is defined as follows:

(4) $$RC=-\overset{T_{2}}{\underset{T_{1}}{\int }}\Delta S_{M}\left(T\right)\mathrm{dT}$$

The temperatures T_1_ and T_2_ are defined by δT_FWHM_ of ΔS_M_(T) peak; as an example, T_1_ = 325 K and T_2_ = 650 K at 1 T. RC reaches 239 J/kg for a magnetic field change of 5 T.

### 3.5. Critical Behavior

The universal behavior of materials can be studied by the critical exponents (β, γ and δ) related to the phase transitions (Table 3). Four distinct conventional models [11] can be used to estimate the critical exponents β, and δ from the M(H) curves such as the: (i) mean field model related to long-range mean field theory, (ii) Heisenberg model correlated to short-range interactions, (iii) 3D-Ising model, and (iv) tricritical mean field model. The exponent β is correlated to the variation of the spontaneous magnetization as a function of temperature (M_S_ ≈ (T − T_C_)^β^). It describes the ordered moment growth for $T<\;T_{C}$; γ is connected to the temperature dependence of the initial magnetic susceptibility against of the temperature ((χ_o_)^−1^ ≈ (T − T_C_)^γ^). It defines the divergence of χ_o_ at T_C_, and δ is associated to with the critical isothermal magnetization. It designates the curvature of the isothermal magnetization curves M(H) at T_C_.

The exponents β, γ and δ have been evaluated by using the modified Arrott plots (MAP) [41], Kouvel-Fisher plots (K–F)( [42,43] and critical isotherm (CI) methods according to evaluated by using Equations:

(5) $$M_{S}\left(T\right)=M_{0}{\left(-\varepsilon \right)}^{\beta };\;\;\varepsilon <0,\;\;T<T_{c}$$

(6) $$\chi_{0}^{-1}\left(T\right)=\left(\frac{h_{0}}{M_{0}}\right)\varepsilon^{\gamma };\;\varepsilon >0,\;\;T>T_{C}$$

(7) $$M=DH^{1/\delta };\;\varepsilon =0,\;T=T_{C}$$

ε = (T-T_C_)/ T_C_ is the reduced temperature; M_o_, h_o_, and D are the critical amplitudes.

**Figure 7:**
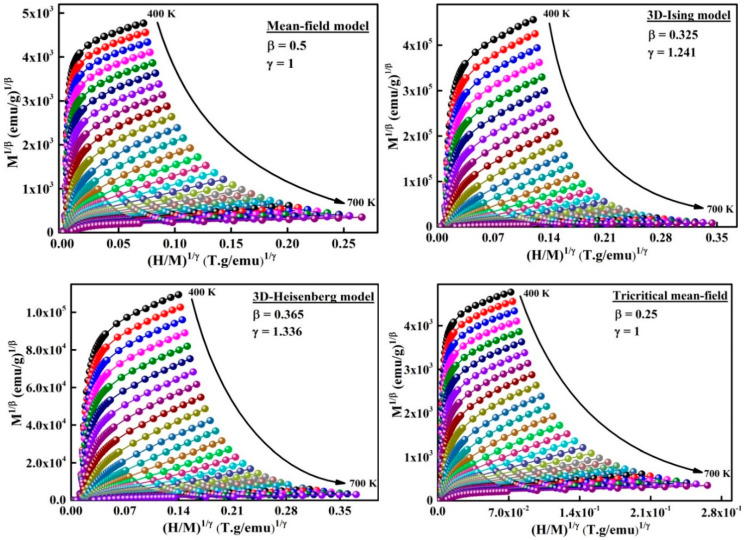
Adapted Arrott plots $M^{1/\beta }\;$ versus $\;\;{\left(H/M\right)}^{1/\gamma }$ from M(H) isotherms.

**Figure 8:**
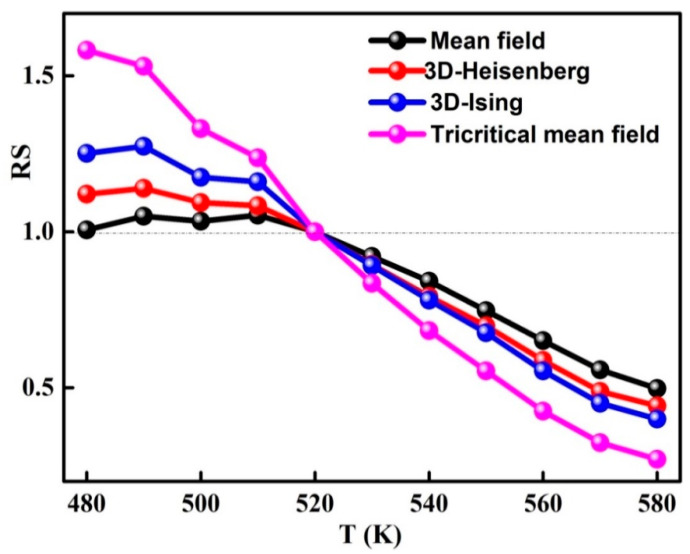
Temperature reliance of the relative slope ($RS=S\left(T\right)/S\left(T_{c}\right))$ for different models.

The modified Arrott plots around $T_{C}$ of the amorphous phase are presented in Figure 7. In order to determine the model that defines the system, it is necessary to evaluate the relative slope $RS=S\left(T\right)/S\left(T_{c}\right)$ which is defined by the relationship between the slope at each temperature T, S(T), and the slope at T_C_, S(T_C_). RS is obtained from the linear fit of the high field area of each curve (Figure 8). Accordingly, the phase transition in the ball-milled Fe_72_Nb_8_B_20_ powders can be described by the mean field model because the relative slope RS is close to the unit.

**Figure 9:**
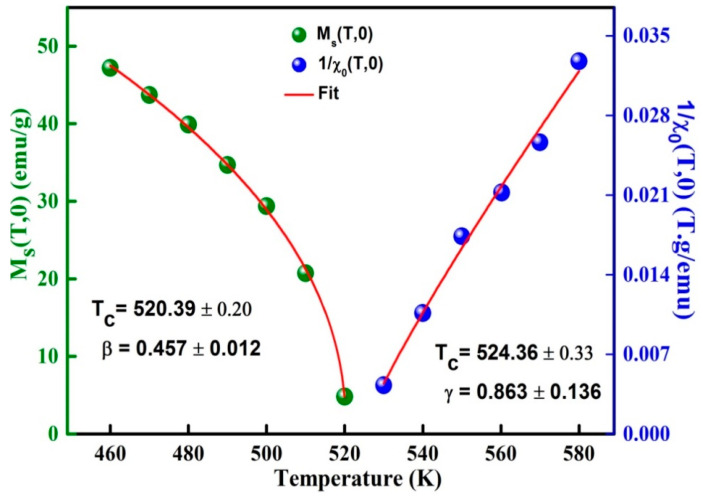
Variation of $M_{S}\left(T,0\right)$ and $\chi_{0}^{-1}\left(T,0\right)$ as a function of the temperature around T_C_.

**Figure 10:**
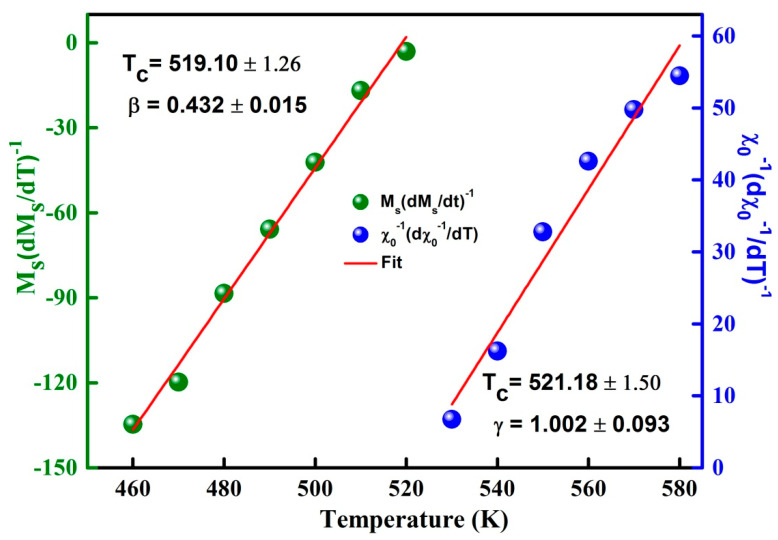
K-F plots of $M_{S}\left(T\right){\left(dM_{S}\left(T\right)/dT\right)}^{-1}$ and $\chi_{0}^{-1}\left(T\right){\left(d\chi_{0}^{-1}\left(T\right)/dT\right)}^{-1}$ versus T.

The exponents β, γ can be deduced by fitting of M_S_(T,0) and $\chi_{0}^{-1}\left(T,0\right)$ curves by means of Equations (5) and (6), respectively (Figure 9). The determined values β = 0.457 ± 0.012 and γ = 0.863 ± 0.136 are reasonable and comparable to those of the mean field (Table 3). $T_{C}$ is approximately 40 K higher than that obtained from the M(T) curve. Those divergences might be correlated to the determination method. Moreover, the Kouvel-Fisher (K-F) method can be used to evaluate the critical exponents’ β and γ from the slopes 1/β and 1/γ of $M_{S}\left(T\right){\left(dM_{S}\left(T\right)/dT\right)}^{-1}$ and $\chi_{0}^{-1}\left(T\right){\left(d\chi_{0}^{-1}\left(T\right)/dT\right)}^{-1}$ plots versus temperature, respectively (Figure 10).

One notes that β = 0.432 ± 0.015 and γ = 1.002 ± 0.093 values are also close to those of the mean field model. Likewise, the Widom scaling relationship permits the determination of the third exponent δ since it is related to β and γ exponent values through the subsequent Equation [44]:

(8) δ = 1 + (γ/β)

**Figure 11:**
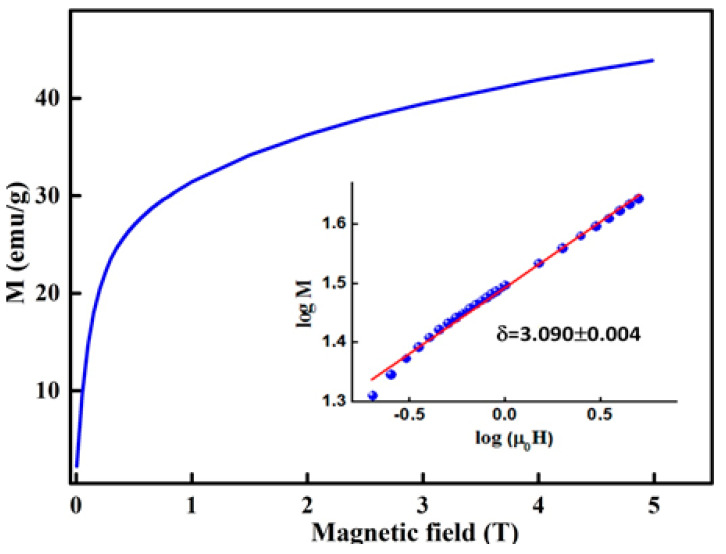
M(H) critical isotherm curve. The *insert* shows the log-log plot.

By using the critical exponents β and γ that are deduced from the K–F method, the obtained δ value, δ = 2.888 ± 0.124 (Table 3), is similar to that estimated from the CI curves (Figure 11), δ = 3.090 ± 0.004. The scaling hypothesis confirms the reliability of the critical exponents and $T_{C}$ [45]:

(9) $$M\left(H,\varepsilon \right)=\varepsilon^{\beta }f_{\pm }\left(H/\varepsilon^{\beta +\gamma }\right)$$

**Figure 12:**
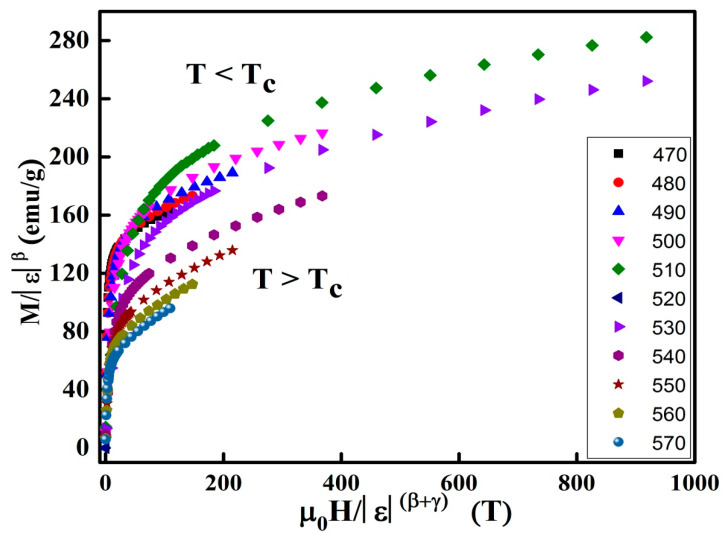
Scaling plots in the vicinity of T_C_.

The regular analytic functions f_+_ and f_−_ are undertaken for T > T_C_ and T < T_C_, respectively. Figure 12 displays the M|ε|^-β^ as a function of H|ε|^-(β+γ)^ are plotted in the vicinity of the T_c_. The accuracy of the predicted critical exponents and $T_{C}$ is confirmed by the presence of two distinct branches below and above TC. 

## 4. Conclusions

Partially amorphous Fe_72_Nb_8_B_20_ powders have been prepared by MA. The MCE, critical behavior, thermal, hyperfine structure and magnetic properties have been investigated. The milling process leads to nanocomposite type structure where nanocrystalline α−Fe(B), Fe(Nb) and Fe_2_B phases and embedded into an amorphous matrix. The detected endothermic and exothermic peaks in the DSC scans are related to the Curie temperature and crystallization of the amorphous phase, respectively. The saturation magnetization and the coercivity increase after the crystallization. The critical exponent’s values (β = 0.457 ± 0.012, γ = 0.863 ± 0.136 and δ = 3.090 ± 0.004) around $T_{C}=480\;K$, are near to those of the mean field model, with a dominating role of magnetic order arising due to long-range ferromagnetic interactions, as the critical exponents are mean-field-like. The maximum entropy change and the refrigerant capacity values are of about 1.45 J/kg·K and 239 J/kg, respectively, for an applied magnetic field of 5 T. These alloys, as magnetocaloric materials, are candidates to work in magnetic refrigeration devices (high temperature span applications) after consolidation in optimized geometries.

## Figures and Tables

**Table 1:** B_hf_, IS, 2ε, QS, Γ and percentage of the formed phases.

Phases	Site	B_hf_ (T) ±0.2	IS (mm/s) ±0.01	2ε or QS (mm/s) ±0.01	Γ (mm/s) ±0.2	Relative area ±1 (%)
α-Fe	SS1	33.0	0.011	−0.016	0.42	12.5
Fe_2_B	SS2	23.2	0.062	0.163	0.60	13.5
Doublet	SS3		−0.203	0.996	0.56	05.0
Amorphous	HFD	10.5	0.281			69.0

**Table 2:** T_C_ and −ΔS_M_(T) in amorphous and partially amorphous (partially am.) Fe-Nb-B alloys.

Composition	Sample Shape	Structure	T_C_ (K)	µ_o_H (T)	-ΔS_M_(T) (J/kg·K)	Ref.
Fe_72_Nb_8_B_20_	Powder	Partially am.	480	2	0.66	This work
Fe_84_Nb_7_B_9_	Ribbons	Amorphous	335	1.5	0.80	[37]
Fe_80.5_Nb_7_B_12.5_	Ribbons	Amorphous	363	0.7	0.72	[18]
Fe_75_Nb_10_B_15_	Powder	Amorphous	250	1.5	0.60	[38]
Fe_79_Nb_7_B_14_	Ribbons	Amorphous	372	1.5	1.07	[17]
Fe_75_Nb_10_B_15_	Powder	Partially am.	395	1.5	0.95	[39]

**Table 3:** Critical exponents of Fe_72_Nb_8_B_20_ powders compared to those of theoretical models. MAP (modified Arrott plot), K-F (Kouvel-Fisher) and CI (critical isotherm).

Model	Technique	β	γ	δ	Ref.
	**MAP**	0.457 ± 0.012	0.863 ± 0.136	2.888 ± 0.124	**This work**
	**K−F**	0.432 ±0.015	1.002 ± 0.093		
	**CI**			3.090 ± 0.004	
Mean field		0.5	1.0	3.0	[11]
3D-Heisenberg		0.365	1.336	4.80	
3D-Ising		0.325	1.241	4.82	
Tricritical mean field		0.25	1.0	5.0	
